# Cardiac Rehabilitation During the COVID-19 Pandemic and the Potential for Digital Technology to Support Physical Activity Maintenance: Qualitative Study

**DOI:** 10.2196/54823

**Published:** 2024-03-14

**Authors:** Linda G Park, Serena Chi, Susan Pitsenbarger, Julene K Johnson, Amit J Shah, Abdelaziz Elnaggar, Julia von Oppenfeld, Evan Cho, Arash Harzand, Mary A Whooley

**Affiliations:** 1 Department of Community Health Systems University of California San Francisco San Francisco, CA United States; 2 Veterans Affairs Medical Center San Francisco, CA United States; 3 Department of Molecular and Cell Biology University of California Berkeley Berkeley, CA United States; 4 Bennett ReGen Dearborn, MI United States; 5 Institute for Health & Aging School of Nursing University of California San Francisco San Francisco, CA United States; 6 Department of Epidemiology Rollins School of Public Health Emory University Atlanta, GA United States; 7 College of Medicine California Northstate University Elk Grove, CA United States; 8 Division of Cardiology, School of Medicine Emory University Atlanta, GA United States; 9 Department of Medicine University of California San Francisco San Francisco, CA United States; 10 Department of Epidemiology & Biostatistics University of California San Francisco San Francisco, CA United States

**Keywords:** cardiac rehabilitation, cardiac rehab, COVID-19, digital health, digital technology, physical activity, physical activity maintenance, social media, older adults, pandemic, social distancing, technology, wearables, CR, exercise, cardiovascular disease, gerontology, geriatric, geriatrics, hospital, medical facility, California, interview, thematic analysis, anxiety

## Abstract

**Background:**

Social distancing from the COVID-19 pandemic may have decreased engagement in cardiac rehabilitation (CR) and may have had possible consequences on post-CR exercise maintenance. The increased use of technology as an adaptation may benefit post-CR participants via wearables and social media. Thus, we sought to explore the possible relationships of both the pandemic and technology on post-CR exercise maintenance.

**Objective:**

This study aimed to (1) understand CR participation during the COVID-19 pandemic, (2) identify perceived barriers and facilitators to physical activity after CR completion, and (3) assess willingness to use technology and social media to support physical activity needs among older adults with cardiovascular disease.

**Methods:**

We recruited participants aged 55 years and older in 3 different CR programs offered at both public and private hospitals in Northern California. We conducted individual interviews on CR experiences, physical activity, and potential for using technology. We used thematic analysis to synthesize the data.

**Results:**

In total, 22 participants (n=9, 41% female participants; mean age 73, SD 8 years) completed in-depth interviews. Themes from participants’ feedback included the following: (1) anxiety and frustration about the wait for CR caused by COVID-19 conditions, (2) positive and safe participant experience once in CR during the pandemic, (3) greater attention needed to patients after completion of CR, (4) notable demand for technology during the pandemic and after completion of CR, and (5) social media networking during the CR program considered valuable if training is provided.

**Conclusions:**

Individuals who completed CR identified shared concerns about continuing physical activity despite having positive experiences during the CR program. There were significant challenges during the pandemic and heightened concerns for safety and health. The idea of providing support by leveraging digital technology (wearable devices and social media for social support) resonated as a potential solution to help bridge the gap from CR to more independent physical activity. More attention is needed to help individuals experience a tailored and safe transition to home to maintain physical activity among those who complete CR.

## Introduction

Cardiac rehabilitation (CR) is a critical aspect of recovery that is offered to adults who experience cardiac events, such as a myocardial infarction, coronary revascularization, and valve replacement, but is significantly underused. CR involves a comprehensive 12-week group program consisting of supervised physical activity training, patient education, and risk modification and is considered an American Heart Association/American College of Cardiology Class IA level recommendation for its health benefits [1]. CR is associated with reduced morbidity and mortality as well as improved quality of life, functional capacity, independence, and symptoms of dyspnea and fatigue [2-6]. Maintaining regular physical activity after CR improves physical function [7,8] and health-related quality of life [4] and is associated with a reduction in the risk of secondary cardiac events, depression, and all-cause mortality [9].

Despite the myriad benefits, many CR participants eventually return to a sedentary lifestyle [10-12] despite the expectation to maintain physical activity independently upon completion. Only 15%-50% report any exercise 6 months after CR completion [10-12], negating the long-term health benefits of CR [13]. Reported barriers to maintaining physical activity after CR include diminished physical condition, competing demands (eg, family health issues), lack of motivation, lack of interest, lack of social support, environmental factors (eg, lack of transportation), and financial costs [14,15]. In addition, participants of CR often receive little to no support during the transition from CR to community- or home-based exercise and desire support mechanisms to ease their transition [16]. The potential enablers to maintaining physical activity after CR include continued contact with CR staff after finishing a CR program, extending the weeks of the CR program, returning for check-ins after CR discharge, having an exercise plan after CR completion, and receiving social support from family and friends [13]. Prior research shows that personal contact is essential to support a successful transition to community-based exercise after CR [16].

In addition to personal contact, the use of personal technology has emerged as a key ally in maintaining an active lifestyle for CR patients. Using technological solutions post phase II CR offers a multitude of benefits for patients. Advanced wearables, such as heart rate monitors and fitness trackers, enable individuals to self-monitor their exercise performance and vital signs, promoting self-awareness and motivation [17]. Mobile apps and telehealth platforms also facilitate access to personalized tracking and provide a digital connection to health care providers [18]. This accessibility enhances patient engagement and adherence to prescribed exercise routines, reducing the risk of relapse and promoting long-term cardiovascular health [19].

The COVID-19 pandemic posed additional challenges related to participation in CR and health-promoting behaviors. A survey study in the United Kingdom reported that COVID-19 lockdown restrictions were associated with significantly decreased participation in CR; changes in CR location, goals, supervision, duration, and enjoyment; and increased perceived effort [20]. A confluence of factors contributed to challenges of participating in CR during the pandemic including programs being forced to suspend or terminate in-person, facility-based services. Declines in CR participation were most marked among dual Medicare and Medicaid enrollees as well as those living in rural areas or socially vulnerable communities based on the Social Vulnerability Index [21]. Compared with CR programs that remained open since the pandemic, the 220 CR centers that closed were more likely to be affiliated with public hospitals located in rural areas and served the most socially vulnerable communities [21].

While mobile phone and social media use has increased among older adults over the past several decades [22], the pandemic showed that older adults rely on and can engage with digital technologies for health care access. Beyond the pandemic, understanding the barriers and experiences of CR participants may lead to more patient-centered CR for those who are unable to or do not wish to participate in facility-based CR and may also be useful for the rapid and effective implementation of CR. This study aimed to (1) describe perceived barriers and facilitators to physical activity after CR completion and (2) understand CR participation during the COVID-19 pandemic among older adults who participated in the ACTION (Americans & Cardiac Rehabilitation Training In Older Adults Needs) study.

## Methods

### Ethical Considerations

The ACTION study was approved by the institutional review boards from all 3 participating sites (John Muir Medical Center IRC-ID 20-08-02; NorthBay Healthcare NBH 21-05; and University of California, San Francisco IRB 20-31215). Participants provided written informed consent by reading the participant information sheet and signing the participant consent form. All participants were given the opportunity to ask questions. All subject data were deidentified (eg, questionnaires, recordings, and transcripts were coded with specific ID numbers). Data were encrypted and stored on a password-secured database. We collected both quantitative and qualitative experiences after completing CR and compensated interview participants US $50 and survey participants US $30. The former will be reported in subsequent publications.

### Design, Recruitment, and Study Sample

This paper describes a qualitative research study that sought to understand beliefs and experiences related to CR among participants with a history of CR participation. Our approach involved conducting in-depth interviews to obtain open-ended responses that were then organized into themes after generating initial codes (ie, thematic analysis). We also collected quantitative data from numerical ratings on comfort levels with various technologies. Two of the coauthors (SP and SC) conducted interviews and data analysis, with a final agreement on the themes by the principal investigator (LGP).

The recruitment sites included 2 community CR centers and 1 university-affiliated center in Northern California. Recruitment occurred between March and September 2022, a time period when public health directives on mask mandates, strict infection control measures, and social distancing practices were evolving from the COVID-19 pandemic. Inclusion criteria were being 55 years of age or older, between 3 and 24 months post-CR participation (of at least 1 session), having English fluency of moderately well to proficient, and being able to provide informed consent. The exclusion criterion was participation in phase III CR (optional extended CR after outpatient CR for those who pay out-of-pocket; reasons for nonparticipation could be the inability to afford phase III CR or other personal reasons). We used multiple methods to identify possible participants. Initial screening for eligibility and recruitment was done by CR staff who also contacted participants to verify eligibility for study participation. We also screened and recruited participants from a pool of respondents who completed a web-based Qualtrics survey that collected quantitative data related to CR experiences, which will be described in future manuscripts. At the end of the survey, respondents indicated if they were interested in participating in an individual 30-minute phone or video interview. Recruitment continued until data saturation was reached when no new codes arose in the analysis of iterative and open-ended questions.

### Data Collection

We collected sociodemographic information such as age, self-identified gender, race, partner status, employment, education, income, and diagnoses for CR. Participants were also asked about their experience with technology. Specifically, they were asked to rate their comfort level with smartphone technology, wearable devices, and social media on a scale of 0-10, with 0 being extremely uncomfortable to 10 being extremely comfortable. The interview guide consisted of 23 questions that were categorized into four major areas: (1) perspectives on their CR experience as a whole, (2) physical activity since completing the CR program, (3) impact of the COVID-19 pandemic on physical activity, and (4) thoughts on various technologies that may aid CR (Multimedia Appendix 1).

Individuals had the choice of interviewing over the phone or video conferencing (ie, Zoom [Zoom Technologies] and Facetime [Apple]); however, all participants elected to be interviewed via phone. All interviews were conducted between June and August 2022 and led by either or both interviewers (SP and SC). All interviews were audio-recorded and transcribed verbatim by a third party. The interviewers also took notes to capture key points and latent data.

### Data Analysis

We analyzed interview transcripts using thematic counts [23] to accurately complete an inductive thematic analysis [24]. Each interview transcript underwent a close reading and coding by 2 raters (SP and SC). The data were organized using Microsoft Excel (Microsoft Corp) and then analyzed [25,26]. Upon reviewing the transcript, 2 raters (SP and SC) independently identified key quotes and developed or assigned them to inductive codes. Such independent analysis ensured intercoder reliability and maintained the credibility and dependability of findings [27,28]. After the initial thematic analysis, raters discussed any coding discrepancies until consensus on the final coding scheme and analysis was achieved or settled by the principal investigator. A running count of responses for each code allowed qualitative data to be transformed for a quantitative understanding of patient responses [29]. Sociodemographic and self-reported technology use were summarized using descriptive statistics.

## Results

### Patient Characteristics and Technology Use

We completed 22 interviews until we achieved data saturation. Table 1 displays the sociodemographic characteristics of the 22 participants, who had a mean age of 73 (SD 8) years, with 41% (n=9) female participants with the majority identifying as White. More than half of the participants were considered low income for living in Northern California. Table 2 outlines the number of participants who were able to complete CR compared to those who had interruptions to CR related to the COVID-19 pandemic.

**Table 1:** Patient characteristics (N=22).

Characteristics		Values, n (%)
Age (years), mean (SD)		73 (8)
Sex (female)		9 (41)
**Race**		
	Asian	2 (9)
	Hispanic	1 (5)
	White	19 (86)
With partner		14 (64)
Employed		6 (27)
College graduate		19 (86)
Income ≥US $75,000 per year		10 (71)^a^
**Diagnoses for CR^b^**		
	Ischemic heart disease	17 (77)
	Heart failure	2 (9)
	Valvular heart disease	3 (14)

^a^A total of 8 participants declined to answer (n=14).

^b^CR: cardiac rehabilitation.

**Table 2:** Participants enrolled in the CR^a^ program between 2020 and 2021.

Timeline (weeks)	Completed CR program (n=16)	CR stopped due to COVID-19 (n=6)
<4	1	0
4-8	3	3
9-12	9	0
≥13	3	1
Unknown	N/A^b^	2

^a^CR: cardiac rehabilitation.

^b^N/A: not available.

The comfort level with technology (smartphone, wearable devices, and social media) was relatively high. Table 2 outlines the number of participants who were able to complete CR compared to those who had interruptions to CR related to the COVID-19 pandemic. Of the 22 participants, 18 owned smartphones, 14 owned a wearable device, and 10 used social media. On a self-reported scale of 0-10 representing the level of comfort using technology, participants reported a mean comfort level of 8.2 with smartphones (n=18), 7.9 with wearable devices (n=14), and 6.8 with social media (n=10).

### Overview of Themes

Five themes prevailed from CR participant responses as displayed in Textbox 1 and as detailed as follows. There was congruence in perspectives without distinct differences noted based on CR site or other patient characteristics. One site had additional challenges beyond the pandemic compared to the other sites due to the relocation of CR services from unexpected facility damage.

**Summary of themes from qualitative interviews.**

**Themes**

- Anxiety and frustration about the wait for cardiac rehabilitation caused by COVID-19 conditions
- Positive and safe participant experience once in cardiac rehabilitation during the pandemic
- Greater attention is needed for patients after cardiac rehabilitation completion
- Notable demand for technology during the pandemic and after cardiac rehabilitation completion
- Social media networking during the cardiac rehabilitation program is considered valuable if training is provided

#### Theme 1: Anxiety and Frustration About the Wait for CR Caused by COVID-19 Conditions

Interviews revealed that participants experienced anxiety and frustration due to the long wait to get into the CR program. The majority of participants (17 of 22) experienced long entry wait periods of between 2 and 3 months to enter the program or faced program closure due to the pandemic. Furthermore, 1 primary on-site location housing the CR program was closed due to facility infrastructure issues, forcing participants to relocate to a more distant location and exacerbating preexisting stressors. In general, delays in getting into the CR program resulted in decreased exercise activity for patients who were newly discharged from the hospital, as they were unable to enter a CR program for many months. Patients expressed their desperation to get into any program, and some called the nearest city with similar programs yet yielded similar wait times. Consequently, patients used resources of personal trainers, internet videos, or were able to get into a home-based physical therapy program. One participant stated:

Yeah, it was a long wait. But I was told, when I started the process, it might be three months before I could get in. At one point I made contact with the program in XX County that’s affiliated with XX to see if I could get in there. And they also had a long wait list.

Facing the COVID-19 barrier, patients expressed they felt an urgency to independently create a healthy environment with exercise and nutrition. It was prevalent with all 22 participants that the urgency to get started after discharge from the hospital was strong. However, without direction or exercise monitoring, patient hesitation to start exercising at home was highly noted, as reflected by 1 patient:

The thing you always worry about when you exercise after having a surgery like that is am I going to have a heart attack.

#### Theme 2: Positive and Safe Participant Experience Once in CR During the Pandemic

Once in the CR program, all of the participants agreed that the program was well organized and professional. After the aggravation of the long wait to enroll in the program for some, patient consensus was that the staff were exceptional in their care and monitoring. One patient explained this:

I was very impressed by both the way in which the program operated, and the people themselves in terms of their competence level and their understanding of the issues, and their concern for patients, and their flexibility.

Many patients considered the program a “safe zone,” which increased their level of confidence to exercise. In developing a fitness regime after cardiac surgery, the presence of monitoring was appreciated:

...knowing there was a registered nurse for every two people, they monitored you – when you’re getting back into physical therapy activity after you’ve had some kind of heart situation, is comforting.

One participant described this further:

The ability to test all the equipment that had monitors was most effective in giving the patients the freedom to try equipment to see what worked well for them to do the work that needed to be done.

Overall, all participants had positive comments about the CR program and felt they would have stayed in the program on a long-term basis if their insurance would cover the cost for more sessions.

#### Theme 3: Greater Attention Needed to Patients After CR Completion

Once patients finished the CR program, many felt on their own and were concerned about keeping up with the regime they learned, as well as lacking the specific equipment provided in the CR program. Voicing concern that there was no follow-up after finishing the program, 1 participant expressed,

Maybe they could have a month later call and say to you how are you doing, are you keeping up with your exercises, how many steps are you having a day?

This quote reflected the sentiment of the majority of the CR program participants. Another said,

I would go back and say “can I use those other four appointments?” because I want to see where I am. I know where I am, and it’s not where I was when I ended the program.

Having no one to follow-up on their progress once home, a participant indicated,

...(her exercise) is on and off. And so, the only consistent exercise is walking. And so, the weights have dropped off pretty much. The bike work has dropped off pretty much, and I’m having trouble now with my back with being able to walk without pain in it. So, I do walk but I don’t walk anywhere near where I’m supposed to be walking as far as the amount of activity I’m supposed to be doing. So, what I planned to do is take my information and start slowly again and build up, pretend I’m at rehab, only it’s just me.

After CR completion, several participants worked and were financially able to hire a personal trainer. Those who worked and did not hire a personal trainer expressed a need to develop greater time management skills to keep up with the needed exercises recommended in the program. Retirees turned to family members and spouses for support with mobile apps, walking together, or going to the community gym.

#### Theme 4: Notable Demand for Technology During the Pandemic and After CR Completion

While on the waitlist for the CR program during COVID-19, many patients sought technological options (eg, computers, phones, and the internet) to sustain physical activity and self-care. A participant said,

Sometimes during Covid, when like everybody was locked down, I did do that on my computer. I did (exercise) classes on my computer.

Most felt it would be a positive alternative if there was another lockdown for those in a program; additionally, 82% (n=18) of the participants believed web-based forms to engage in physical activity would be a great option once they finished in the program.

Participants were asked their opinion about the idea of using a wearable device for self-monitoring to help with physical activity after the prescribed CR program (with a prompt related to having a personal coach). The reassurance of a monitor in tracking several health metrics along with personal progress and having a personal coach to review health data were considered most important to individuals who had experienced a life-threatening event. Having these resources would give them a sense of being in a “safe zone.” One participant’s comment reflected similar responses from the other participants:

I’m through the program, but now I have to pay and if there was something like this, you know, with a wrist monitor and you could do your exercises and meet with, you know a personal coach from the rehab, or who’s monitoring you, that would be wonderful.

Finding the resources on “what to do, how to use and what information can I get from this tool” was a key factor in moving forward working with a utility device.

In total, 14 participants stated they owned a wearable device; however, not all participants actively used it. The barrier to use (brought up by 4 participants) was the lack of user friendliness or guidance in learning how to use the device. Some participant quotes were “I can see what I want to see, I can probably learn more,” “It’s manageable, but I feel my abilities with it are limited,” and “I have a smartwatch, but I need help setting it up.” When considering using a wearable device for tracking health metrics and monitoring personal progress, a concern arose that “I would need training on how to set it up.” A participant considered seeking assistance from family: “my grandchildren would help my wife. They were born with those things, you know?”

#### Theme 5: Social Media Networking During the CR Program Considered Valuable if Training Was Provided

When asked about their opinion on using social media to supplement their CR experience, half of the participants voiced interest in using social media to see what others were experiencing while undergoing the program or to build camaraderie with other CR participants, especially in the context of limited social interaction during the COVID-19 pandemic. During the height of the pandemic, social distancing precautions prevented CR participants from being able to talk with other attendees and develop friendships with people having similar issues. With reference to having an opportunity to join a Facebook private group with CR participants*,* someone stated:

that would be ideal. Yes. I think this would be ideal because, you know, there's always questions that come up, I think, you know, are you going through this right now or, you know, all this is suddenly affecting me.

The abilities to support one another and share information were both key components in participants’ interest in using Facebook groups. A participant’s perspective on its value was to “establish a good rapport with some of these folks in the social media, you become the coach to each other.”

Most of the participants were open to trying technology, including Facebook, with the condition there was training or assistance. With social media, some responded they did not know how to maneuver through Facebook beyond keeping up to date with family or browsing posts. A participant shared, “I would do it probably. If I could work the Facebook system to do that, I would.” Ultimately, there was agreement on the need for technological options and guidance while in a CR program as well as after finishing the program.

## Discussion

### Principal Findings

This qualitative study presents the positive experiences participants have in CR, the impact of the COVID-19 pandemic on both physical activity and their CR experience, and the need for additional clinical and social support after CR completion (eg, wearable devices and social media). Along with the 5 themes that have been presented, the findings could also be summarized from the standpoint of stages of CR. More specifically, the way in which technology could be deployed as a solution to barriers at all stages. Before the CR initiation, there was heightened anxiety in getting enrolled into a program due to long pandemic-related waitlists and program closures. During this waiting period, participants expressed fear and hesitation to start exercising without supervision. Technology in the form of positive messages and education regarding safe exercise could be delivered before, during, and after CR to address these concerns. Due to the persistence of long wait times for CR initiation after the pandemic, clinicians may reconsider the role of proper discharge instructions from the hospital and include more details about exercise safety while they wait for phase II CR. During enrollment in CR, there was an increase in participant confidence and comfort with exercise. During and after CR participation, patients reported the potential benefit of wearable health devices to track exercise and to have those data reviewed by their CR team. In addition, patients were enthusiastic about using wearable devices and health apps to support their participation in phase II CR or their health behaviors after completing CR. Furthermore, wearable devices could potentially provide a practical alternative to CR in the case where in-person CR is inaccessible (ie, long enrollment waitlists or another pandemic). After CR, participants expressed concerns about maintaining exercise without equipment and not being able to ask questions to a CR provider. This older group of participants were open to participating in social media networking groups as a means to increase social support and use peers as a resource for answering questions. Some participants cited they could solicit technology support from their family members if needed. These data affirm the benefits of CR, despite pandemic-related barriers, and the positive outlook on using technology as a solution to shortcomings in exercise monitoring and social support.

Although drop-offs in exercise following CR completion are well-known, our study found several reasons that may help design future policy changes or interventions that can increase post-CR exercise. As noted above in theme 3, after program completion, patients had variable access to necessary exercise equipment and felt a lack of guidance or follow-up from professional staff. Such findings are key for understanding the impacts on long-term physical and mental health. Exercise is critical for maintaining cardiovascular health and reducing the risk of future cardiac events [30], thus policy changes including reimbursement of long-term exercise equipment (eg, stationary bike) or periodic web-based or in-person check-ins with the CR team could be considered. With limited access to exercise equipment or continued training, patients may struggle to maintain their physical health improvements. Additionally, lack of check-ins following CR completion decreases adherence to a healthy lifestyle and increases the likelihood of patients returning to previous unhealthy behaviors such as smoking, unhealthy eating habits, or a sedentary lifestyle, thereby increasing the risk of future cardiac events and negative patient outcomes [31]. In addition to physical effects, the period following CR may have a significant psychological impact as patients may experience depression, anxiety, or fear of future cardiac events [32]. While CR may assist patients in managing these emotions, without continued support, patients may struggle to cope. Evident in our study, patients felt ill-prepared to maintain health improvements and track progress, heavily relying on guesswork with high anxiety. This only magnifies existing mental health risks. Thus, attention to post-CR health remains an area for improvement for health care providers. Further research is needed to determine optimal check-in frequency and methods for ongoing physical and psychological support.

This study specifically addressed the potential role of integrating technology to improve physical activity after CR completion. Traditionally, older adults have been considered resistant to the use of new technologies; however, our study refutes this myth and validates that older adults are eager to engage with certain technologies and have high self-reported comfort levels with using technology. Other recent studies have shown that modern technology, including wearable devices and mobile apps, can be practical tools in maintaining physical activity levels after completing CR [33,34]. In particular, older adults have demonstrated a strong potential for adopting new technologies to support their physical activity and maintain healthy lifestyles [35,36]. For example, in a previous study, we found that a home-based CR program that included wearable activity trackers and web-based support was well received by older adults who reported improved physical activity levels and overall satisfaction with the program [37]. Other studies have found that technology-based interventions can be effective in reducing anxiety and depression in post-CR patients, as well as improving adherence to healthy lifestyle behaviors [38,39]. With the increasing availability and affordability of digital health technologies, there is significant potential to integrate these tools into CR programs to provide ongoing support and improve long-term health outcomes for patients. However, it is crucial to consider older adults’ specific needs and preferences in designing and implementing technology-based interventions and provide appropriate training and support to ensure successful adoption and sustained use.

There are several clinical and research implications that can be derived from this study’s findings. CR providers should assess participant needs early on in their program to assess psychological status (anxiety or depression) and other barriers (equipment or gym access) to be successful with independent physical activity after CR completion. In addition, providers can assess other needs such as health conditions that require safety precautions for home exercise. Standardized evidence-based practice guidelines are needed to guide participants who graduate from the CR program. Patients need to be taught about long-term habit formation and motivation to continue physical activity after phase II CR is over, as well as discuss a transition plan in advance of finishing the programs with their CR clinical team. In addition, incorporating the use of a wearable device with personal monitoring could alleviate fears of having secondary events as expressed by many participants. Building in the use of digital technology during phase CR II may be helpful so patients have a warm-up period with support for using the same technology independently after CR completion. For research implications, opportunities include collecting and analyzing data on long-term clinical outcomes (rehospitalization and mortality) from diverse populations who receive any versus no support after CR ends. There is also a need for conducting cost analyses on tools such as digital wearable devices and mobile apps to improve health outcomes.

There are also significant policy implications. This study emphasizes the interest of older adults to engage with wearable devices and social media and may be relevant to multiple stakeholders (ie, payors and health systems) in making decisions on what to pay for and how to deploy the technology. Participants’ expressed need for additional support after phase II CR to maintain physical activity is also an important policy implication. Extending insurance coverage for maintenance of remote home-based CR services beyond the traditional 12-week program will help participants transition to independent exercise. This covered extension could be in the form of phase III CR or different payment models that fuse remote patient monitoring with coaching for long-term exercise maintenance. Based on this study, the integration of wearable device data into these services may be beneficial, and financial reimbursement and secure implementation remain an area for future investigation.

### Limitations

Our sample mostly comprised White individuals and those from higher educational backgrounds; thus, the findings may not be generalizable to other diverse racial groups and those with low educational attainment. We are also unclear whether financial resources were associated with CR attendance among our participants, including ownership of smartphones and wearable technology that may have influenced their opinions about technology use. In addition, we recruited all participants from 3 urban institutions in Northern California; therefore, our sample may not be generalizable to a broader population including rural populations. There may be heterogeneity in the participants’ experiences of older adults and their CR experiences with at least 1 completed CR session (versus up to 36 sessions in some programs) within 3-24 months after CR participation. Despite these limitations, this study provides important insights into the lived experiences and perspectives of 22 older adults representing 3 different CR programs. This study confirmed previous research that describes the perceived lack of support after CR termination [14]. In addition, this study supports the perceived benefits of adding digital technology as a component of providing tailored feedback to CR participants.

### Conclusions

Despite the critical role physical activity plays in sustaining patient cardiovascular health improvements, maintaining adequate activity levels after CR proves to be an immense challenge for several reasons. For example, the transition from the supportive and structured environment of CR centers to daily life leaves patients without the guidance and encouragement they once had. This study highlights requests from participants for regular check-ins and support from health professionals, as well as the integration of digital technologies to improve individuals’ motivation and accountability in adhering to their exercise routines. Amplified by the COVID-19 pandemic, these challenges demand thoughtful consideration and tailored strategies to ensure sustained adherence to regular physical activity. This study’s findings support the opportunity to leverage technology through wearable devices or mobile apps to sustain engagement in healthy lifestyle behavior because they are cost-effective, tailored, and provide motivation and support for patients in the long term. Although these data were collected during a pandemic, the experiences and perspectives of the participants are generalizable in the current environment of CR with the need to support patients after CR completion and the opportunities that technology offers.

## Appendix

Multimedia Appendix 1 Interview guide.

## Abbreviations

ACTION: Americans & Cardiac Rehabilitation Training In Older Adults Needs
CR: cardiac rehabilitation

## Data Availability

The qualitative data generated and analyzed during this study are available from the corresponding author upon reasonable request.
