# Supplementary material for: Cardiac Rehabilitation During the COVID-19 Pandemic and the Potential for Digital Technology to Support Physical Activity Maintenance: Qualitative Study
Source: JMIR Cardio. 2024 Mar 14;8:e54823. doi: 10.2196/54823 (PMC10941834; doi:10.2196/54823)
Supplement: Multimedia Appendix 1 [file cardio_v8i1e54823_app1.docx]

**Interview guide**

Thinking about the cardiac rehabilitation (CR) program as a whole,

1. What did you like most about the CR program?
2. What did you like least about the CR program?
3. What was the most useful about the program?
4. What was the least useful?
5. What things would you like changed about the program?
6. Can you describe any system/facility barriers to participating in the program? (delayed start, staffing shortages, Covid-related facility shut-down)
7. Can you describe any personal barriers to participating in the program? (personal illness, financial constraints, work, family)
8. Can you discuss any changes specific to your cultural needs that would make your CR experience better? (prompts: language barrier, interactions with staff, variety of exercise)

Since completing the CR program,

1. How much exercise are you recommended to do weekly? And how much do you actually do?
2. What are your typical exercise activities?
3. Is there anything you would change in your typical day and week to include more physical activity?
4. If applicable, what do you need to be more successful in exercising the recommended amount?
5. What did you learn from CR that you are applying after you completed the program?
6. Are there resources you need to be more successful in your exercise program?

Due to the COVID-19 pandemic,

1. How has your physical activity changed as a result of the pandemic?
2. How has your attitude about physical activity changed as a result of the pandemic?
3. a) "Do you have a smart phone?"; b) "Do you have a wearable device for physical activity?"; and c) “Do you use social media (e.g.. Facebook, Instagram)?”
4. If YES to a-c in #17, on a scale from 0 to 10, how comfortable are you with using the features of: a) smart phone, b) wearable device, d) social media (10 being extremely comfortable). Please explain.
5. What is your opinion about participating in a home-based CR program that uses phone or video to help your physical activity DURING the prescribed CR program? (personal coaching and monitoring)
6. What is your opinion about participating in a home-based CR program that uses phone or video to help your physical activity AFTER the prescribed CR program? (personal coaching and monitoring)
7. What is your opinion about using a wearable device to help your physical activity DURING the prescribed CR program? (personal coaching and monitoring)
8. What is your opinion about using a wearable device to help your physical activity AFTER the prescribed CR program? (personal coaching and monitoring)
9. What is your opinion about using social media (Facebook private groups) to interact with other participants DURING the prescribed CR program?
10. What is your opinion about using social media (Facebook private groups) to interact with other participants AFTER the prescribed CR program?
